# Distribution, fractionation and sources of rare earth elements in suspended particulate matter in a tropical agricultural catchment, northeast Thailand

**DOI:** 10.7717/peerj.10853

**Published:** 2021-02-24

**Authors:** Kunhua Yang, Guilin Han, Jie Zeng, Wenxiang Zhou

**Affiliations:** Institute of Earth Sciences, China University of Geoscience (Beijing), Beijing, China

**Keywords:** Rare earth element, Suspended particulate matter, Agricultural catchment, Water/particle interaction, Mun River, Thailand

## Abstract

Forty-eight suspended particulate matter (SPM) samples were collected from the Mun River, northeast Thailand and its junction with the Mekong River, to investigate the relationship between the distribution of rare earth elements (REE) in SPM and the soils in the watershed. The total REE contents (∑REE) in SPM in the Mun River ranged from 78.5 to 377.8 mg/kg with the average of 189.3 mg/kg, which was lower than ∑REE of 222.3 mg/kg at the Mekong River (one sample at junction). The Post Archean Australia Shale (PAAS)-normalized ratios of light REE (LREE), middle REE (MREE) and heavy REE (HREE) were averaged to 1.0, 1.3 and 1.0, which showed a clear enrichment in MREE. In short, along the Mun River, the REE contents in SPM were decreasing, and the PAAS-normalized patterns of REE showed gradually flat. The REE content in SPM and soils are highest in the upper catchment, indicating that soil/bedrock is the most important source of REE in SPM. Additionally, the positive Eu anomaly was enhanced by the higher Ca content in SPM (*R* = 0.45), which may be caused by more feldspars or carbonates with Ca and Eu substituting Ca. The results present the REE behaviors of SPM in the Mun River and relationship between REE in SPM and soil/bedrock, the findings may support the other studies in catchment weathering.

## Introduction

The rare earth elements (REE) are a collection of fifteen trace elements: La, Ce, Pr, Nd, Pm, Sm, Eu, Gd, Tb, Dy, Ho, Er, Tm, Yb and Lu (i.e., “the lanthanides”), all of which (except Pm) be found together in the geological materials. The REE are usually divided into three groups as light REE (LREE) (La–Nd), middle REE (MREE) (Sm–Dy) and heavy REE (HREE) (Ho–Yb). The REE have common trivalent oxidation state so they are an extremely coherent group in aspect of chemical behaviors, for which REE have been used to investigate the weathering processes and water-rock or water-particle interactions (Han & Liu, 2007). In most cases, concentrations of REE in environmental materials such as sediments are related to their parent materials (Kritsananuwat et al., 2015). For example, the abundances of REE in coals vary under complex deposited environments and epigenetic evolution (Dai et al., 2012).

In the aqueous environment, REE are sensitive to the physicochemical properties (pH, redox potential, salinity), the concentration of complexing agents, secondary minerals and clay minerals, which control REE behaviors in the water/particle interaction (e.g., adsorption/desorption reactions) (Bayon et al., 2015; Gwenzi et al., 2018). In solid phases, positively charged REE complexes and ions are strongly adsorbed by secondary minerals which are characterized by high surface areas like Al/Fe/Mn oxides/oxyhydroxides (Gosselin et al., 1992), and the total adsorption increases when the pH and organic matter increase (Wen et al., 2002). In general, particulate and colloidal phases in many rivers are the major loads of REE, which tends to be enriched in the light REE and/or the middle REE (Åström & Corin, 2003). Suspended particulate matter (SPM) typically refers to the fine particulate matter with a diameter of more than 2 μm, which is held in all streams under natural conditions. The REE in SPM may account for about 35%-95% of the total flux of REE in rivers (Da Silva et al., 2018), which have various sources that are different in background contents of REE, weathering and transport. One of the primary sources of SPM is the erosion of soils and the re-suspension of sediment (Liu & Han, 2020; Roussiez, Aubert & Heussner, 2013), making their REE contents easy to be impacted by soils and bedrocks (Jones et al., 2016). For instance, more abundant REE in SPM are found in rivers draining igneous and metamorphic terranes than volcanic or sedimentary rocks (Bayon et al., 2015). Thus, the knowledge of particulate REE provides information relevant to natural weathering erosion and river transport (Cholet et al., 2019).

The Mun River drains in extensively cultivated areas, where few studies have reported the concentrations and behaviors of REE in different natural materials, except several recent studies focusing on soils and basalts (Yuan et al., 2019; Zhou et al., 2020). Moreover, REE geochemistry in the tropical agricultural watershed systems have rarely been reported, especially to examine the impact factors of REE in SPM (Comte et al., 2012; Davis et al., 2017). The REE may be a useful tool to track the geochemical processes of SPM and to link the geochemistry of REE and environment factors like soils in the Mun River catchment. Therefore, the goals of this study are: (1) to examine the REE contents in SPM in each reach of Mun River; (2) to investigate the REE composition in SPM and its spatial variation; (3) to identify the relation between REE in SPM and REE in soil/bedrock; (4) to evaluate the flux of REE in SPM in the Mun River to the Mekong River in the dry season. This study provides more information to integrate a view at hydrological processes and soils erosion in a tropical agricultural watershed.

## Materials and Methods

### Geological background

The Mun River is the second longest river in Thailand and the longest river in the western branches of the Mekong River. With an area of about 71,000 km^2^, the Mun River catchment covers the northeast Thailand from 14°05′ N to 16°25′ N and from 101°15′ E to 105°40′ E. The Mun River extends about 900 km and is often divided into three stretches by longitudes of 102.5°E and 104.5°E. Although the maximum elevation is 1,070 m above sea level (asl) in southwest basin, the average elevation of the Mun River basin (MRB) is about 195 m asl (Yang & Han, 2020).

**Figure 1:**
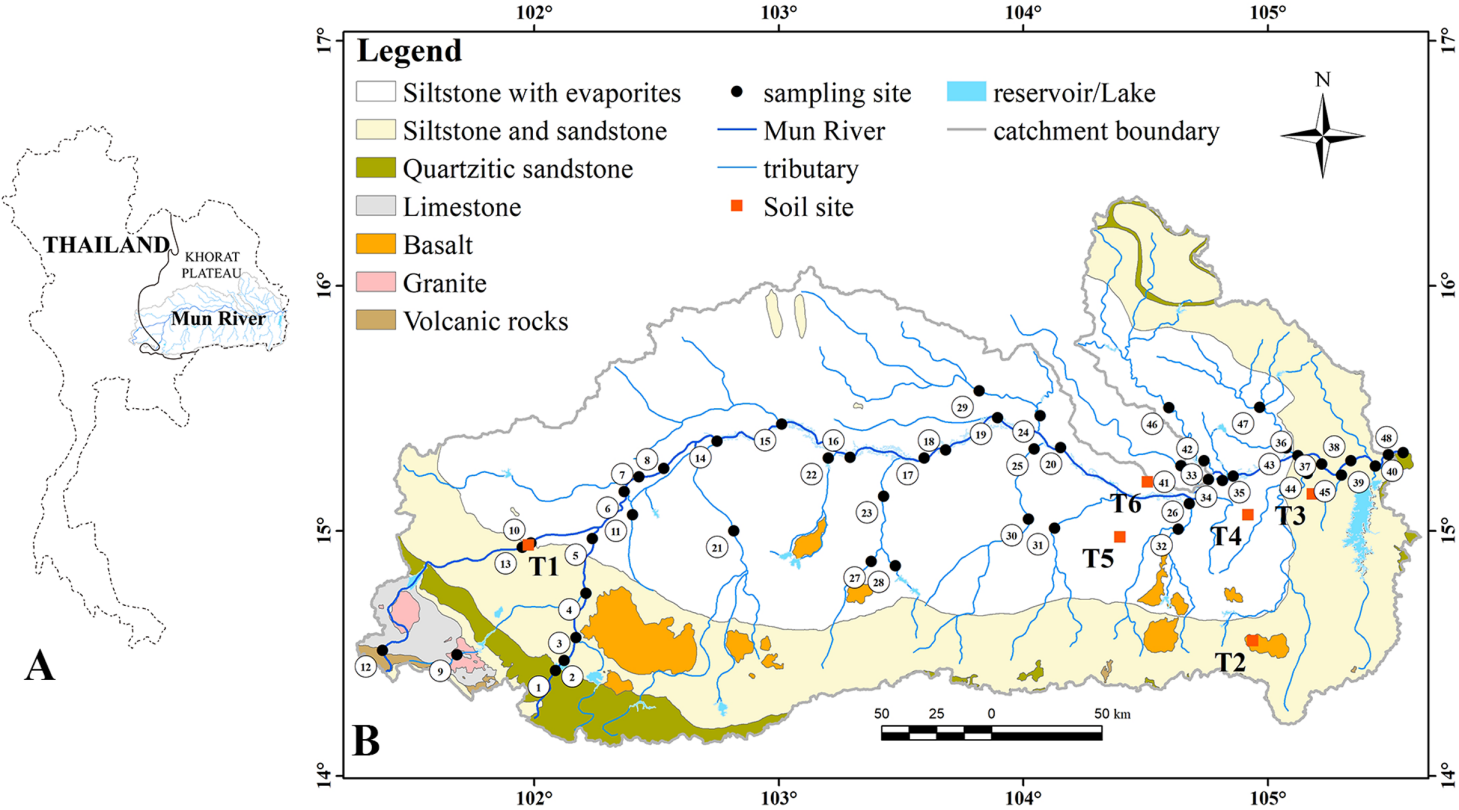
Location of the Mun River, sampling sites and lithology map (A) Location of the Mun River. (B) Location of sampling sites and lithology map of the Mun River catchment.

With respect to tectonic division, the MRB is located in the Khorat Plateau Basin (Fig. 1A). Intensive rural activities are held in the Mun River, and paddy fields account for about 75% of the total cropping land area, which accounts for over 50% area of the MRB (Prabnakorn et al., 2018). The paddy fields are mainly located on the vast floodplain of the MRB. The total rainfall and discharge are very low in the dry season compared to the wet season, the contamination can be amplified in the Mun River. In the dry season, most rainfall occurred in the mountain area, and the evaporation was significantly high in the plain area (Yang & Han, 2020). The irrigated area/total area was highest in the upper reaches, while it was similar between the middle reaches and the lower reaches (Prabnakorn et al., 2018). The population and number of industries were highest in the middle reaches, following by the upper reaches and then the lower reaches (Akter & Babel, 2012; Li et al., 2019).

The lithology of the study area was described in the previous literature (Yang & Han, 2020) and the details were as follows. The siltstone with evaporites, which include carnallite, gypsum, and tachyhydrite, is the dominant rock type and is distributed across the vast central and northern basin. In the western and southern of MRB, siltstone, sandstone and quartzitic sandstone are unevenly distributed. The weathering intensity of different rocks contributes to the landform of the MRB. In addition, Jurassic volcanic rocks and granites are distributed sporadically in the western MRB, while basalt occurs in the southern MRB. The granites mainly refer to the biotite granites, and the basaltic rocks contain the minerals including olivine, clinopyroxene and plagioclase.

### Sampling strategies and analytical methods

Considering the land use, lithology and hydrology, 48 SPM samples were collected on 5–28 March 2018 (the dry season). Compared to the wet season, the dilution effect of rainwater on river are negligible in the dry season Han et al. (2019), due to lower rainfall and discharge. A total of 48 sampling sites were chosen (Fig. 1B), which samples from 1 to 13 were located in the upper reaches; from 14 to 32 in the middle reaches; from 33 to 47 in the lower reaches and sample 48 was collected at junction of the Mun River and the Mekong River that was considered as the sample of the Mekong River water. River water was taken from bank or middle channel on the bridges in sampling depth of ca. 0.5 m. A 10% v/v nitric acid-cleaned LDPE bag was used to collect river water. The water samples were filtered through 0.22 μm cellulose acetate membranes, while the SPM were left on the filter membrane. In the field, pH and oxidation-reduction potential (ORP) were determined using a YSI multi-parameter meter, and the location data were recorded by GPS.

The SPM were digested using the method according to Tang & Han (2017) with slight modification, and the procedure was described in the previous study (Zeng, Han & Yang, 2020). The SPM were dried in an oven at about 55 °C and weighed to calculate the concentration of SPM. Then, chemical digestions of SPM and digested procedure blank were accomplished in a super-clean laboratory (positive-pressure filtered air lab), which is a metal-free class 1,000 (ISO class 6) clean laboratory at the Surficial Environment Geochemistry Laboratory, China University of Geosciences (Beijing). Ultrapure water with an 18.2 MΩ/cm resistivity (Cascada, Pall, Westborough, MA, USA) and sub-boiling high-purity reagents were used. Firstly, about 100 mg of SPM sample powders were digested using a mixture of 1 ml ultrapure hydrofluoric acid (HF) and 3 ml ultrapure nitric acid (HNO_3_) in a sealed polytetrafluoroethylene beaker and heated on a hotplate at 140 °C for 6 h. Second, after cooling and drying, 2 ml ultrapure HNO_3_ were added into the sealed beaker and heated at 120 °C for 3 h (this step conducted two times). Third, the completely digested samples were dried again and re-dissolved using 100 ml 3% v/v HNO_3_ in the 10% v/v nitric acid-cleaned HDPE bottles. Finally, the digested samples were transferred into 7 ml centrifuge tubes for element analysis.

The major metal concentrations (Fe, Mn, Al, Ca, Mg, K and Na) in SPM were analyzed by inductively coupled plasma optical emission spectroscopy (ICP-OES, Optima 5300DV; PerkinElmer, Waltham, MA, USA). The data quality was monitored using duplicates, method blanks and standard reference materials (GBW(E)081531) from the Chinese Academy of Measurement Sciences. The REE concentrations were determined by inductively coupled plasma mass spectrometry (ICP-MS, ELAN DRC-e; Perkin Elmer, Waltham, MA, USA). The standard solutions (ICP-MS-68A; High-purity Standards Inc., North Charleston, SC, USA), which contained all REE at 10 mg/L in 2% HNO_3_, were diluted to 1, 10, 100 μg/L with 2% HNO_3_ to calculate the calibration curve. A 1% v/v HNO_3_ solution was used to flush the introduction system before every measurement to avoid the memory effect. The following masses of REE were used in the ICP-MS measurement: ^139^La, ^140^Ce, ^141^Pr, ^142^Nd, ^152^Sm, ^153^Eu, ^158^Gd, ^159^Tb, ^164^Dy, ^165^Ho, ^166^Er, ^169^Tm, ^174^Yb, and ^175^Lu. Samples were initially diluted 1: 10 with Rh/Ir internal standard (10 mg/L Rh and Ir in 2% HNO_3_). Oxide mass interferences were minimized based on Ce/CeO ratios (Xu, Morgan & Rate, 2018). The procedural blanks were less than 5% for La, 10% for Ce and 3–5% for other REE of the samples when compared to the lowest REE concentration. The measurement accuracy was ±3% for HREE and ±5% for LREE related to the quality control standards. Above elements analyses were taken at the Institute of Geographic Sciences and Natural Resources Research, Chinese Academy of Sciences, China.

The Cerium (Ce) and the Europium (Eu) anomalies were calculated as follows (Han et al., 2009):

(1) $$\rm Ce\ anomaly = 2 \times (Ce_{sample}/Ce_{PAAS})/(La_{sample}/La_{PAAS} + Pr_{sample}/Pr_{PAAS})$$

(2) $$\rm Eu \ anomaly = 2 \times (Eu_{sample}/Eu_{PAAS})/(Sm_{sample}/Sm_{PAAS} + Gd_{sample}/Gd_{PAAS})$$

The positive and negative Ce anomalies and Eu anomalies are defined as the values of >1 and <1, respectively.

The data of REE in soils quoted from the previous study, and the information of six soil profile location and REE analysis can be found in Zhou et al. (2020). The dissolved organic carbon (DOC) concentrations of river water quoted from Liu et al. (2019).

## Results

### Physicochemical properties of river water and SPM

**Figure 2:**
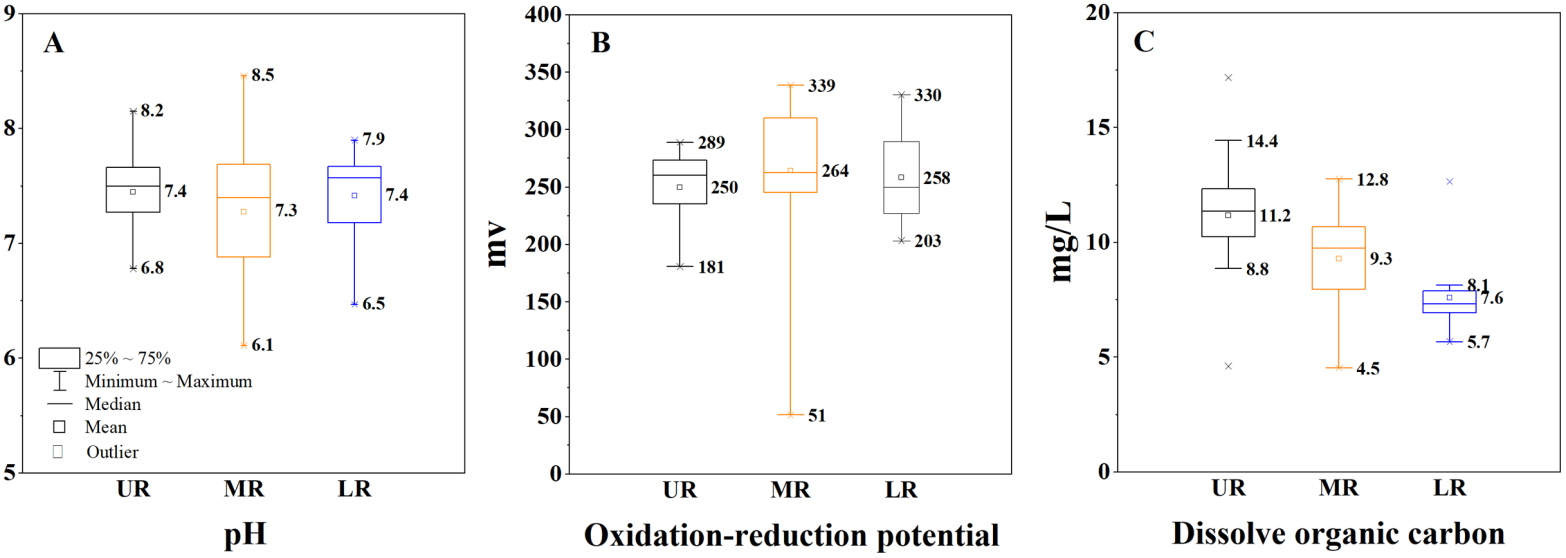
Variation of pH, oxidation-reduction potential (ORP) and dissolved organic carbon (DOC)of river water. (A) Variation of pH. (B) Variation of ORP. (C) Variation of DOC. UR indicates the upper reaches, MR indicates the middle reaches, and LR indicates thelower reaches.

The physicochemical properties of the river water (pH, ORP and DOC) were presented in Fig. 2. The data of DOC were from Liu et al. (2019). The pH values ranged from 6.1 to 8.5 with the mean value of 7.4 in the Mun River, and the average of pH was 7.4, 7.3 and 7.4 in the upper, middle and lower reaches, respectively. The ORP values ranged from 51 to 339 mv with the mean value of 258 mv in the Mun River, and the average of ORP was 250, 264 and 258 mv from the upper to lower reaches. The DOC values ranged from 4.5 to 17.2 mg/L with the mean value of 9.3 mg/L, and the average DOC was 11.2, 9.3 and 7.6 mg/L in the upper, middle and lower Mun River, respectively. In the Mekong River, pH, ORP and DOC were 7.6, 173 mv and 2.6 mg/L, respectively. Therefore, ORP and DOC were higher in the Mun River, while the pH showed higher value in the Mekong River.

**Figure 3:**
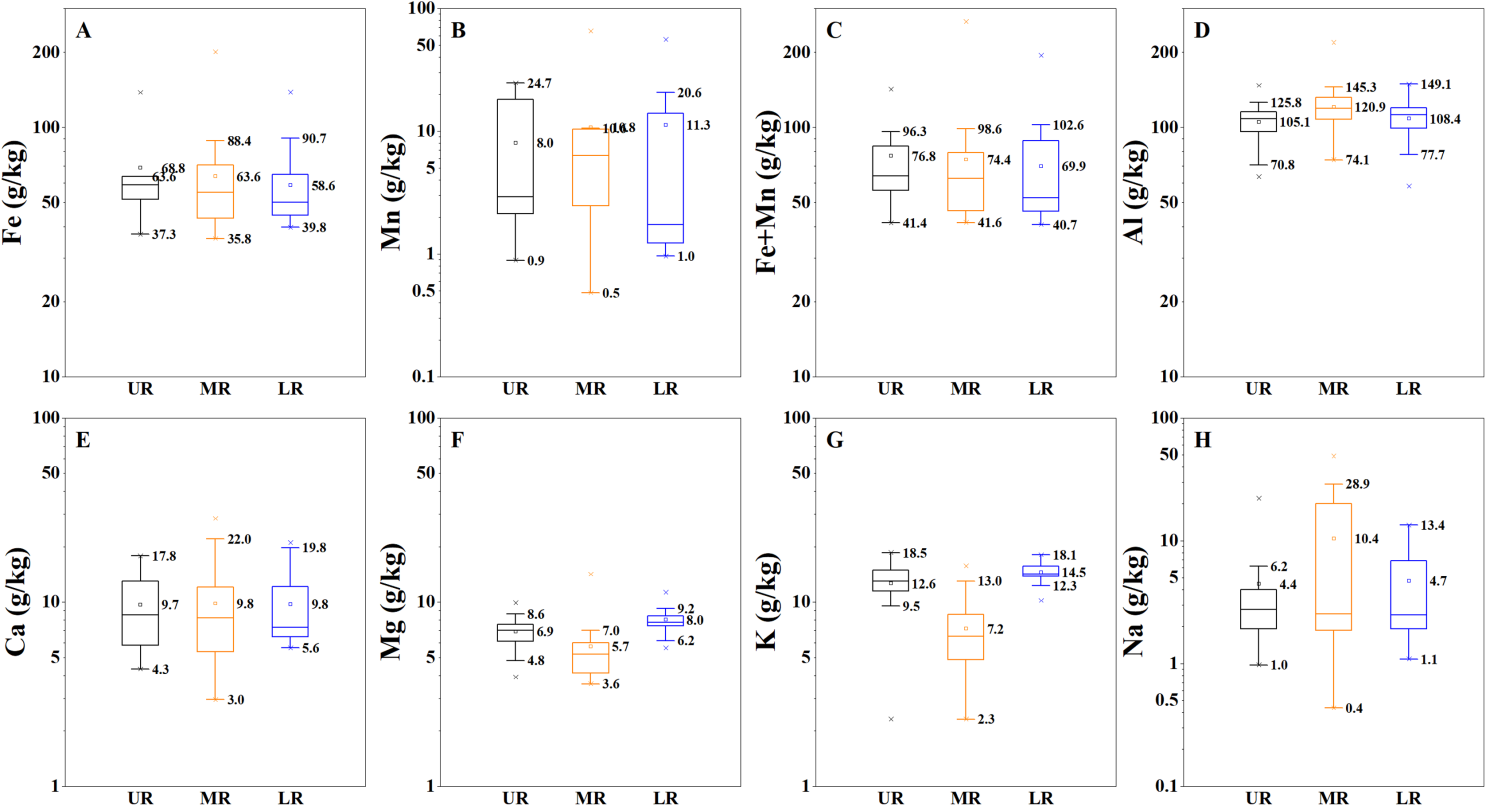
Variation of metal contents (Fe, Mn, Al, Ca, Mg, K and Na) of SPM. (A) Variation of Fe. (B) Variation of Mn. (C) Variation of Fe+Mn. (D) Variation of Al. (E) Variation of Mg. (G) Variation of K. (H) Variation of Na. UR indicates the upper reaches, MR indicates the middle reaches,and LR indicates the lower reaches.

The contents of metal elements (Fe, Mn, Al, Ca, Mg, K and Na) in SPM were given in Fig. 3. The average of Fe, Mn, Al, Ca, Mg, K and Na contents was 61.8, 9.2, 113.3, 9.5, 6.7, 10.9 and 6.8 g/kg in the Mun River, while it was 53.6, 1.5, 55.0, 116.2, 3.3, 8.6, 21.2 and 2.4 in the Mekong River. On the one hand, the metal contents show a trend as Al > Fe > K > Ca > Mn > Mg > Na in the Mun River, and the contents of metal elements are higher in the Mun River than those in the Mekong River, except the Al, K and Mg. Additionally, as for spatial trend, Al and Na are similar (highest in the middle reaches), Mg and K are similar (lowest in the middle reaches), Fe and Mn are opposite (tends to decrease or tends to increase in downstream), and Ca shows weak spatial variation. As the two of main factors impacting REE behaviors, the Fe and Mn contents are concerned primarily in this study. The Fe contents in SPM varied from 35.8 to 200.4 g/kg, and the average of Fe contents was 68.8, 63.6 and 58.6 g/kg in the upper to lower reaches. The Mn contents in SPM varied from 0.5 to 65.2 g/kg in the Mun River, and the average was 8.0, 10.8 and 11.3 g/kg in the upper to lower reaches.

### Characteristics of REE contents in the SPM

**Figure 4:**
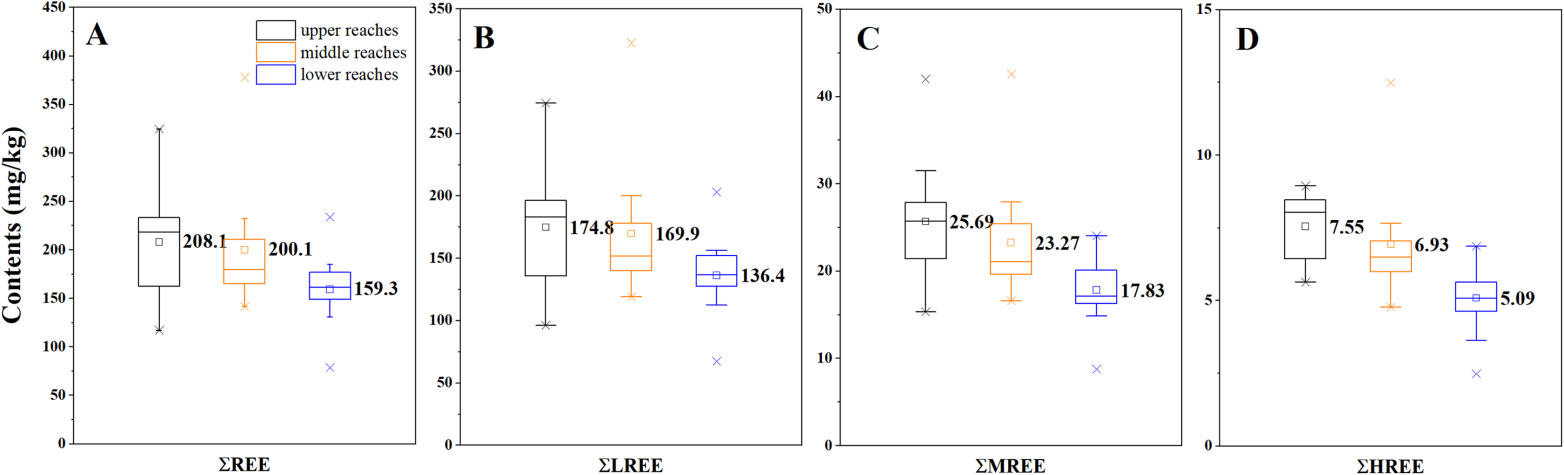
Box diagrams of ∑REE, ∑LREE, ∑MREE and ∑HREE in the SPM in the upper, middle and lower reaches of the Mun River. (A) Variation of ∑REE. (B) Variation of ∑LREE. (C) Variation of ∑MREE. (D) Variation of ∑HREE.

In the Mun River, total REE contents (∑REE) in the SPM ranged from 78.5 to 377.8 mg/kg with the mean value of 189.3 mg/kg, which was higher than those of the average upper crust (146.4 mg/kg) and was lower than those of the Mekong River (222.3 mg/kg, at the junction in this study) (Taylor & McLennan, 1985). Among the total REE, LREE accounted for 77.0%~87.5%, MREE accounted for 11.11%~21.63% and HREE accounted for 2.53%~6.30%. As exhibited in Fig. 4 and Table 1, the order of the mean values and median values of ∑REE was decreasing along the spatial transect from the upper reaches to the lower reaches, and the same trends were shown in the ∑LREE, ∑MREE and ∑HREE. In general, the relative contents of REE (mg/kg) in the SPM are decreasing from upstream to downstream of the Mun River in the dry season.

**Table 1:** The REE contents of the SPM in the upper, middle and lower reaches of the main stream and tributaries of the Mun River.

	∑REE	∑LREE	∑MREE	∑HREE	∑LREE/∑HREE	∑LREE/∑MREE	∑MREE/∑HREE
	mg/kg						
UR^a^	208.05	174.82	25.69	7.55	23.2	6.8	3.39
MR^b^	200.14	169.95	23.27	6.93	24.4	7.3	3.35
LR^c^	157.68	136.38	17.83	5.09	26.8	7.7	3.47
M-UR^d^	217.1	184.1	25.73	7.20	25.3	7.2	3.52
M-MR	203.0	172.0	23.92	7.12	24.1	7.1	3.37
M-LR	161.4	137.9	18.16	5.40	25.7	7.6	3.37
L^e^	170.7	143.5	20.90	6.31	23.8	7.0	3.38
R^f^	197.7	168.4	22.65	6.60	25.6	7.4	3.44
L-UR	202.7	167.2	26.92	8.54	19.7	6.1	3.16
R-UR	157.5	130.8	20.35	6.36	20.6	6.4	3.20
L-MR&LR	152.5	130.0	17.47	5.04	26.2	7.5	3.50
R-MR&LR	201.0	171.6	22.84	6.62	26.0	7.5	3.46

**Notes:**

a UR refers to the upper reaches.

b MR refers to the middle reaches.

c LR refers to the lower reaches.

d M-UR refers to the upper reaches of the main stream.

e L refers to the tributaries in the left branch.

f R refers to the tributaries in the right branch.

In the tributaries, the average REE contents showed higher values in the upper reaches of the left branch and the middle and lower reaches of the right branch (Table 1), where the granites and basalts were widely distributed (Fig. 1), indicating the REE contents in the SPM of tributaries draining granites and basalts were higher than those draining sedimentary rocks.

## Discussion

### Normalized REE to post archean Australia shale and soil

Comparing different parts of the Mun River in Table 1, it can be found that the lowest LREE/HREE fractionation was recorded in the tributaries of the upper reaches. For further study of REE’s fractionation, the REE contents in SPM were normalized. Since the primary lithology was the sedimentary rocks in the study area, the Post Archean Australia Shale (PAAS, (Taylor & McLennan, 1985)) was used to calculate the normalized values (Table 2). Further, the ∑REE contents in soils followed as upper reaches>middle reaches>lower reaches.

**Table 2:** REE contents of the post-Archean Australian shale (PAAS) (Taylor & McLennan, 1985) and the soils in the Mun River catchment (Zhou et al., 2020).

	La	Ce	Pr	Nd	Sm	Eu	Gd	Tb	Dy	Ho	Er	Tm	Yb	Lu	∑REE
PAAS	38.2	79.6	8.8	33.9	5.55	1.08	4.66	0.77	4.68	0.99	2.85	0.41	2.82	0.43	184.8
Soil-UR^a^	42.3	85.8	9.4	34.1	6.76	1.51	6.17	1.02	5.57	1.15	3.25	0.54	3.38	0.52	201.4
Soil-MR^b^	16.7	33.0	3.6	13.4	2.5	0.5	2.2	0.4	2.1	0.5	1.3	0.2	1.4	0.2	78.0
Soil-LR^c^	10.5	17.6	2.6	10.4	2.0	0.5	1.8	0.3	1.7	0.3	1.0	0.2	1.0	0.2	50.1
Soil-Avg.^d^	17.9	34.1	4.1	15.3	3.0	0.7	2.6	0.4	2.5	0.5	1.5	0.2	1.5	0.2	84.6

**Notes:**

a Soil-UR refers to the soil in the upper reaches.

b Soil-MR refers to the soil in the middle reaches.

c Soil-LR refers to the soil in the lower reaches.

d Soil-Avg. refers to the average of soils in the Mun River catchment.

**Figure 5:**
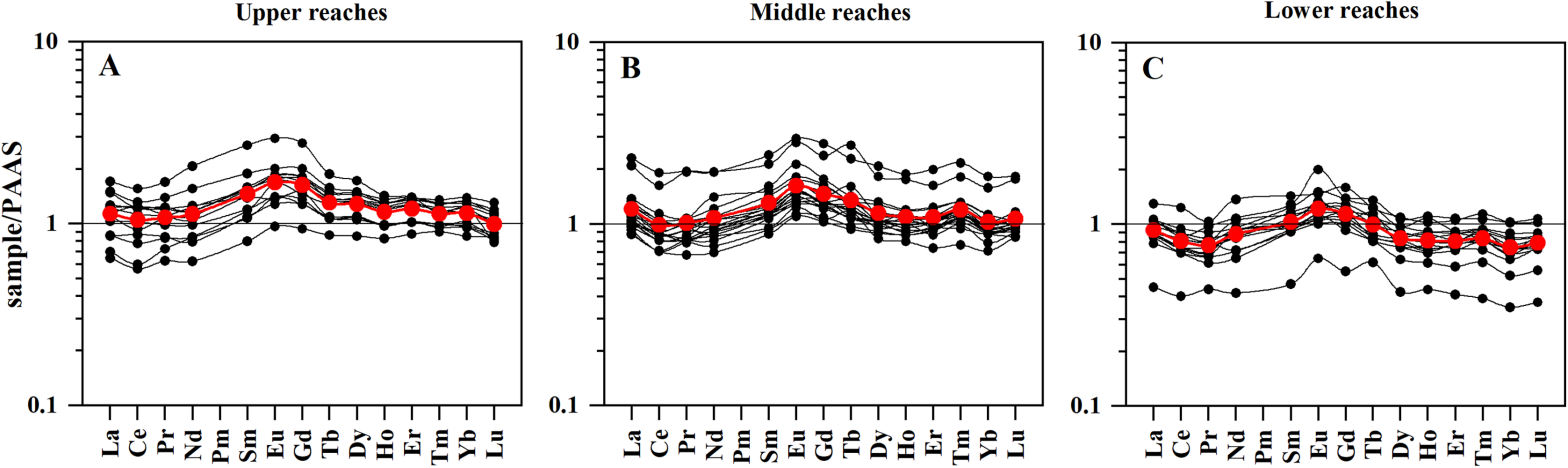
PAAS-normalized REE patterns of SPM in the upper reaches, in the middle reaches and in the lower reaches. (A) PAAS-normalized REE patterns of SPM in the upper reaches. (B) PAAS-normalized REE patterns of SPM in the middle reaches. (C) PAAS-normalized REE patterns of SPM in the lower reaches. Red lines indicate theaverage of normalized patterns.

**Figure 6:**
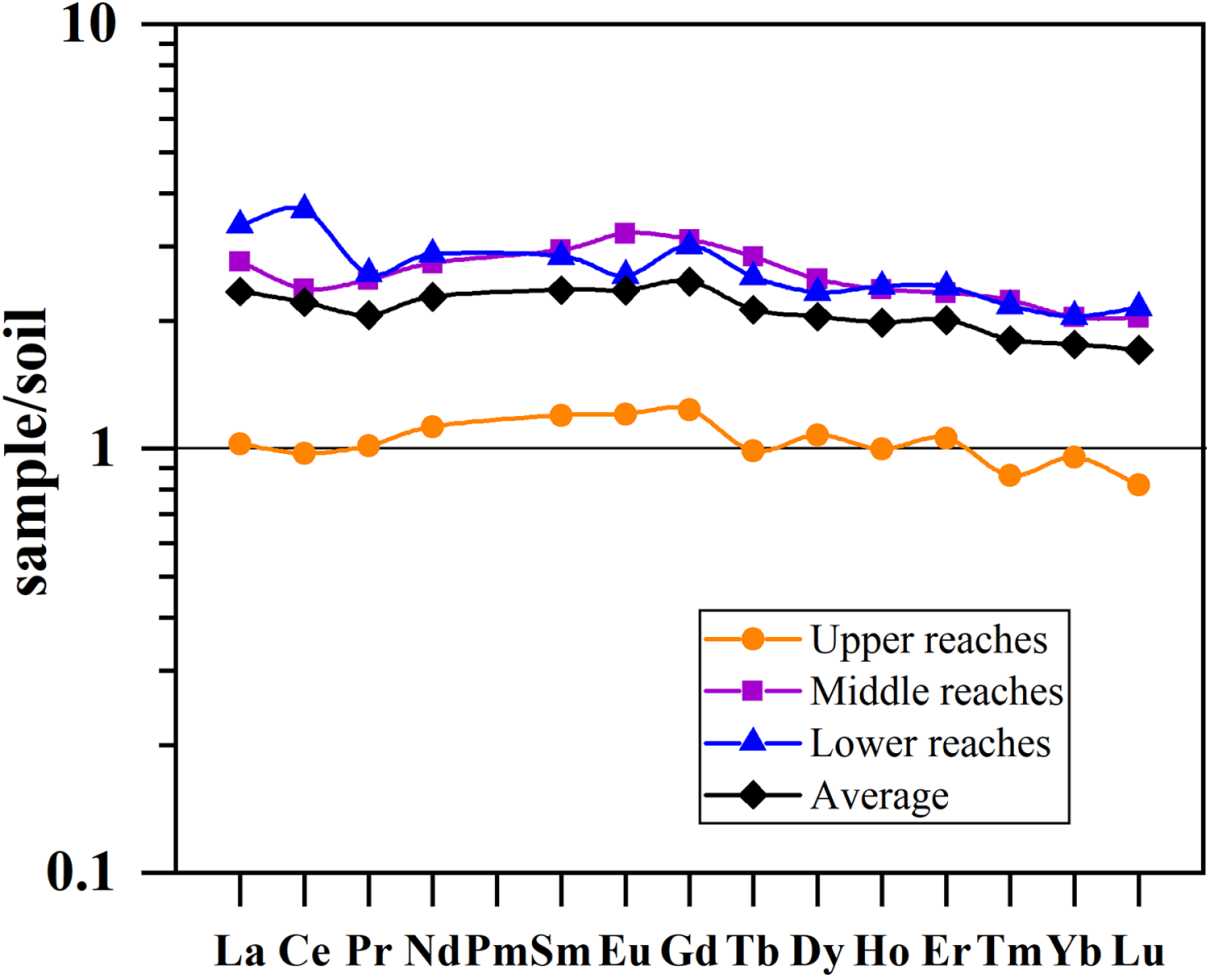
REE fractionation patterns normalized to soil in the Mun River Basin. The calculation uses REE data of SPM and soil in each reach, respectively.

The PAAS-normalized ratios of LREE varied from 0.4 to 2.0 with the mean value of 1.0, those of MREE varied from 0.5 to 2.4 with the mean value of 1.3, and those of HREE varied from 0.4 to 1.9 with the mean value of 1.0. In other words, the PAAS-normalized REE patterns of SPM mainly showed a marked enrichment in MREE (Fig. 5), and the average of normalized patterns was different in the upper, middle and lower reaches (red lines). In the upper reaches, average PAAS-normalized REE pattern showed obvious MREE enrichment and slight HREE enrichment. In the middle reaches, average PAAS-normalized REE pattern only showed MREE enrichment. In the lower reaches, it obviously turned to slight LREE and HREE depletion. In general, the PAAS-normalized REE patterns of SPM gradually changed from MREE enriched in the upper reaches to relatively flat in the lower reaches. In other words, in the fractionation processes of REE, the MREE and HREE were more efficiently removed than the LREE in the SPM of the Mun River. In order to compare the REE of SPM and soils, the soil-normalized REE patterns were displayed in Fig. 6. In the upper reaches, SPM showed obvious enrichment in Nd, Sm, Eu and Gd compared with soils, and contents of other elements (La-Pr and Tb-Lu) were similar with soils.

### Ce and Eu anomalies

**Figure 7:**
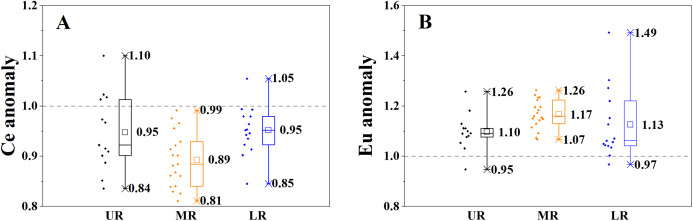
The spatial variation of Cerium (Ce) and Europium (Eu) anomalies of the SPM. (A) Spatial variation of Ce anomaly. (B) Spatial variation of Eu anomaly. UR indicates the upper reaches, MR indicates the middle reaches, and LR indicates the lower reaches. ORP indicates the oxidation-reduction potential. DOC indicates the dissolve organic carbon. MEC indicates the metal elements content, including Al, Fe, Mn, Ca, Mg, K and Na. The delta Ce indicates the Ce anomaly. The delta Eu indicates the Eu anomaly.

Due to the special electron configuration in the outer shell and different valence states from other REE, Ce and Eu can occur as Ce (IV) and Eu (II). The Eu anomaly is strongly lithology-dependent (Petrosino et al., 2013), while the Ce anomaly is related to either lithology or physicochemical parameters, such as pH and ORP (Han et al., 2009). Most of SPM in the Mun River showed negative Ce anomalies and positive Eu anomalies (Fig. 7). The Ce and Eu anomalies were changing along the flow path, illustrating that the sources of SPM and the water quality maybe changed spatially. The Eu anomalies of SPM may be related to the parent samples (rocks/soils). Eu^2+^ always take place of Ca^2+^ in plagioclase during magmatic processes, due to their similar ionic radius (Alderton, Pearce & Potts, 1980). Ca content in SPM was averaged to 11.3 g/kg in the left branch, may relating to distribution of limestones in the upper reaches, while that was 9.0 g/kg in the right branch. In the Mun River, Eu anomalies showed a positive correlation with Ca in SPM (*R* = 0.452, *p* < 0.01). That is, higher Ca in SPM is likely an important factor leading to positive Eu anomalies. Therefore, the physicochemical properties of river water should be considered as the primary controlling factors in Ce anomalies of SPM, whereas the Eu anomalies have been more influenced by the parent samples (bed rock and soils). In the middle and lower reaches, the REE contents in SPM were distinctly higher than those of soils, and SPM showed the enrichment in LREE and MREE instead of HREE compared to soils. The negative Ce anomaly and positive Eu anomaly were shown in the middle reaches, and the positive Ce anomaly and negative Eu anomaly were shown in the lower reaches. The soil-normalized REE patterns suggest that the REE contents in SPM were related to the soils.

### Implications for river environmental management

#### Local environmental effects

Since the discharge of the river was low in the dry season (low dilution effect of rainwater), the impacts of human activities may be amplified in the Mun River. The distribution of REE of SPM in the Mun River was distinctly similar to the soils, indicating that the inputs from soils can cause the significant impacts on the river water, especially the agricultural pollution in the upper reaches.

On the other hand, more attention should be paid in REE behaviors of SPM in the wet season, owing to over 40% of soils suffered from erosion (Akter & Babel, 2012). Moreover, there is more erosion of soils contributing to the SPM in the middle and lower reaches within the larger rainfall amount. Therefore, it can be inferred that the REE contents in SPM are higher in the wet season than those in the dry season (Suja, Fernandes & Rao, 2017). Moreover, the pH of river water was lower in the wet season (Liang et al., 2019). Thus, the contents of poisonous REE (such as Gd and Tb) into the dissolved fraction could be enhanced because of the preferred removal.

#### REE in SPM flowing into the Mekong River

The total suspended sediment was about 2.9 × 10^7^ ton (t) during the dry season (2017–2018) at the hydrological station located at outlet of the Mun River (Li et al., 2019). In this study, the content of ∑REE in SPM was 161.2 mg/kg in the Mun River (no. 40 sample) flowing into the Mekong River (no. 48 sample, 222.3 mg/kg). Thus, the ∑REE in SPM of about 4,675 t flowed out to the Mekong River during the dry season (November–April). Meanwhile, in SPM, the ∑LREE, ∑MREE and ∑HREE were 3,970 t, 543 t and 162 t, respectively. The percentage of MREE was higher in the SPM in the Mun River. The pH was higher, and the ORP, DOC were lower in the Mekong River. Therefore, it is reasonable to infer that more MREE of SPM could be preferentially removed into the Mekong River water.

## Conclusions

This study analyzed the distribution and fractionation of the REE of the SPM in each reach of Mun River and its junction with the Mekong River, and discussed the potential links between SPM and soil/bedrock. Among the REE (78.5–377.8 mg/kg), contents were ordered as: ∑LREE > ∑MREE > ∑HREE. The REE contents in SPM were higher in the tributaries that drain granites and basalt rocks (right branches) than sediment rocks (left branches). PAAS-normalized REE patterns of SPM showed gradually flat along the river flow path, with the marked enrichment in MREE. In the upper reaches, average PAAS-normalized ratios of LREE, MREE and HREE in SPM were 1.1, 1.45 and 1.16 in the upper reaches, 1.0, 1.27 and 1.02 in the middle reaches, 0.9, 1.01 and 0.78 in the lower reaches. Soil-normalized patterns of SPM exhibited the highest enrichment of REE in the upper reaches, and Eu anomaly showed positive correlation with Ca content. Thus, the REE in SPM potentially presented the links between SPM and soil/bedrock. In the future work, it may be necessary further comprehensive studies on behavior of REE within the Critical Zone system, particularly on the environment-human pathway and biological uptake.

## Supplemental Information

10.7717/peerj.10853/supp-1 Supplemental Information 1 The REE and metal contents of suspended particulate matter (SPM), statistic values of ∑REE, ∑LREE, ∑MREE and ∑HREE and pH, ORP and DOC of river water.

## Additional Information and Declarations

### Data Availability

The following information was supplied regarding data availability: Raw data are available as a Supplemental File.
